# Feeding native rumen microbial supplements increases energy-corrected milk production and feed efficiency by Holstein cows

**DOI:** 10.3168/jdsc.2022-0210

**Published:** 2022-05-21

**Authors:** A.M. Dickerson, F. Yang, H.B. Green, M.M. Embree, J.K. Drackley

**Affiliations:** 1 Department of Animal Sciences, University of Illinois, Urbana-Champaign 61801; 2 Native Microbials, San Diego, CA 92121

## Abstract

The objective of this study was to investigate the effects of 2 novel native microbial feed supplements (MFS) on milk yield, milk composition, dry matter intake (DMI), body weight (BW), body condition score (BCS), and efficiency. A total of 73 lactating Holstein cows were enrolled over 4 mo. During a 2-wk covariate period, baseline data were collected. Using a randomized complete block design, cows were blocked in groups of 3 by days in milk (DIM), production, and parity. Within blocks cows were randomly assigned to a basal diet with a 150 g/d ground corn topdress and no MFS (control), the basal diet supplemented with a 5 g/d dose of *Clostridium beijerinckii* ASCUSDY20 and *Pichia kudriavzevii* ASCUSDY21 in a carrier mixed with a 150 g/d ground corn topdress (MFS1), or the basal diet with a 5 g/d dose of *Ruminococcus bovis* ASCUSDY10, *Butyrivibrio fibrisolvens* ASCUSDY19, *Clostridium beijerinckii* ASCUSDY20, and *Pichia kudriavzevii* ASCUSDY21 in a carrier mixed in a 150 g/d ground corn topdress (MFS2) for 140 d. Cows were fed the diets as a total mixed ration (TMR) for ad libitum DMI once daily. Analysis showed numerical increases in performance variables such as milk yield and fat and protein contents in both MFS1 and MFS2, although the differences were not significant over time. For energy-corrected milk (ECM), other solids percentage, and BW there were significant treatment by time interactions. Cows fed MFS1 and MFS2 produced 1.4 kg/d and 1.6 kg/d more ECM than control cows, respectively (time-dependent treatment significance). Up to wk 13 of treatment, both MFS groups had higher ECM production than the control group but after this point MFS1 rapidly decreased to the same level as control. All cows displayed a gradual decrease in ECM after wk 13 but the MFS2 group remained greater than both control and MFS1. A similar trend in the content of other milk solids was observed. Cow BW was affected over time; both control and MFS2 cows gained weight at a similar rate throughout the study, whereas the weight gain of MFS1 cows decreased briefly from wk 13 to 17 before increasing again. Feed efficiency tended to be improved by MFS, with greater improvement by MFS2 than by MFS1. The DIM when the supplementation began had a significant impact on MFS2 efficacy, where the supplementation of native rumen microbes at an earlier DIM resulted in a greater gain in ECM over the 140-d trial. A similar trend was observed for MFS1, although the effect was not significant. Our results suggest that dairy cow lactation stage and energy requirements also play an important role in product efficacy.

## Highlights

• Supplementation with native rumen organisms improves energy-corrected milk production
• The 4-microbe supplement performed better than the 2-microbe supplement
• No negative impact on health or body weight from supplementation
• Production improvements may be influenced by lactation stage

## Graphical Abstract

**Figure:**
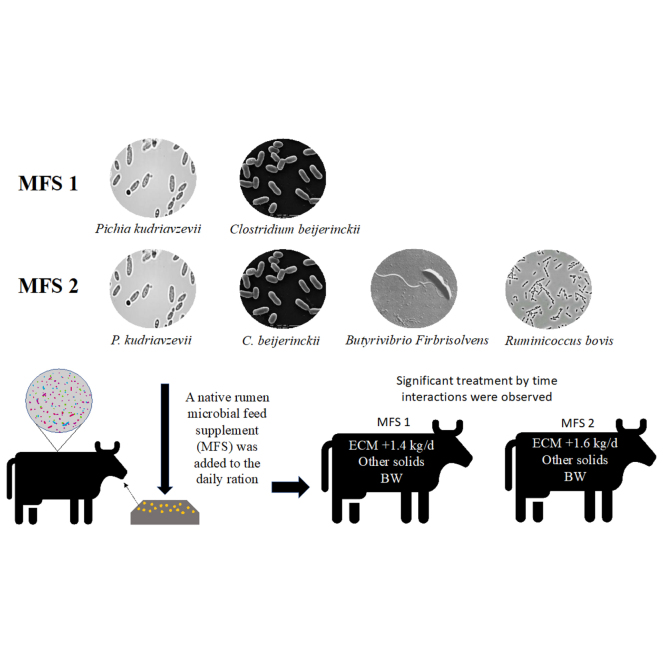
**Summary:** This study tested the effect of 2 microbial feed supplements consisting of microorganisms sourced from the rumen on production of Holstein dairy cows. Supplementation improved energy-corrected milk production, and the response to the 4-microbe supplement was greater than the 2-microbe supplement. Greater production improvements occurred in cows that started receiving microbes earlier in lactation, especially in the group receiving the 4-microbe supplement.

Improving productivity and feed efficiency while minimizing environmental impact has been a growing goal of the dairy industry in recent years. The complex microbiome of the rumen plays a key role in a cow's ability to obtain nutrients and produce milk. In the rumen, various species of microorganisms digest plant materials via fermentation and produce VFA that can be used by the animal. The primary end products produced from fermentation are acetate, propionate, and butyrate, as well as carbon dioxide and methane gases (Bergman, 1990).

Unlike conventional direct-fed microbials, microbial feed supplements (**MFS**) are composed of microorganisms that are native to the rumen environment and positively associated with healthy and productive dairy cows (Zengler and Embree, 2016). These microorganisms digest different feed ingredients and produce VFA that can be used by the cows for milk production. For example, *Butyrivibrio fibrisolvens* has long been known to ferment plant structural carbohydrates and produce stearic acid (C18:0), which is used in the synthesis of milk fat (Loften et al., 2014), via the biohydrogenation of linoleic acid (Kepler et al., 1966). *Ruminococcus bovis* is a novel species recently isolated from healthy cow rumen contents, which can ferment a variety of carbohydrates, including starch, to produce acetate as a major end product (Gaffney et al., 2021). Rumen acetate is a major energy source and precursor for milk fat synthesis. An increase in rumen acetate has been associated with increased milk fat yield in Holstein cows (Urrutia et al., 2019). *Pichia kudriavzevii* has high cellulase activity and can degrade cellulose and hemicellulose to monosaccharides, which can be used by other rumen microorganisms (Akita et al., 2021; Suntara et al., 2021b). *Pichia kudriavzevii* increased DM digestibility of rice straw (Suntara et al., 2021a). *Clostridium beijerinckii* forms a cooperative relationship with other complex carbohydrate degraders to produce hydrogen, as well as acetate and butyrate (Zhang et al., 2012; Gomez-Flores et al., 2017). Similar to acetate, an increased rumen butyrate concentration is positively correlated with milk production in dairy cows (Huhtanen et al., 1993; Seymour et al., 2005). Together, these native rumen microorganisms can act to improve feed digestibility and milk production.

A previous study from Iowa State University explored the use of an MFS (**MFS1**) containing *C. beijerinckii* and *P. kudriavzevii* in lactating Holstein cows and its impact on milk production. The authors found a trend for increases in feed efficiency (**FE**), milk yield, and ECM in cows with MFS supplementation when compared with control cows, although the overall increases were not significant (Goetz et al., 2021). Cows were 119 DIM at the start of the study, with the result that possible effects in early lactation might have been missed. Because of this and the differences noted with production level, it was of interest to gain further information about MFS1. Furthermore, we speculated that efficacy of MFS1 could be improved by adding *R. bovis* to aid starch fermentation and *B. fibrisolvens* for additional CHO breakdown on its own as well as providing substrates for cooperative fermentation with the organisms in MFS1. Therefore, in the present study, we evaluated the effect of 2 MFS combinations, MFS1 (Galaxis, Native Microbials) and **MFS2** (Galaxis Frontier, Native Microbials), on milk yield, milk composition, DMI, BW, BCS, and FE of lactating Holstein cows.

All procedures were approved by the University of Illinois Urbana-Champaign Institutional Animal Care and Use Committee (protocol #20197). The experiment was conducted from October 27, 2020, to July 20, 2021. Cows were blocked by DIM, production, and parity, then were randomly assigned to 3 treatment groups within blocks. The treatment groups were control (no MFS supplementation), MFS1 (consisted of 2 × 10^7^ cfu/g of *P. kudriavzevii* ASCUSDY21 and 2 × 10^6^ cfu/g of *C. beijerinckii* ASCUSDY20), and MFS2 (consisted of 2 × 10^7^ cfu/g of *P. kudriavzevii* ASCUSDY21, 2 × 10^6^ cfu/g of *C. beijerinckii* ASCUSDY20, 2 × 10^7^ cfu/g of *R. bovis* ASCUSDY10, and 2 × 10^7^ cfu/g of *B. fibrisolvens* ASCUSDY19). To detect statistically significant results for a 5% increase in milk production, a power analysis determined that a minimum of 24 cows per treatment group (total 72 cows) were required. A total of 73 Holstein cows owned by the University of Illinois were enrolled between October 27, 2020, and February 16, 2021. Cows ranged from 43 to 145 DIM. Of the 73 cows, 8 were replaced due to low production or health concerns and 3 were removed later in the study without replacements. Specifically, 1 cow from the control group died after 8 wk in the study due to toxic mastitis, 1 cow from the MFS2 group died after 9 wk in the study due to toxic mastitis, and 1 cow from the MFS2 group was euthanized at 8 wk in the study due to injury.

The study consisted of a 2-wk covariate period as baseline and a 140-d treatment period. All cows received a TMR once daily at 0900 h and had ad libitum access to water and feed. The diet (Table 1) consisted of corn silage, alfalfa silage, alfalfa hay, canola meal, corn gluten feed pellets, ground corn, and a vitamin and mineral premix to meet or exceed the predicted energy, protein, mineral, and vitamin requirements (NRC, 2001). The amount of TMR offered was adjusted daily based on the previous day's average DMI with a target of <10% refusals. Extra feed was given as needed during the evening herd checks and recorded. The control group received a 150 g/d ground corn topdress. For the MFS1 and MFS2 group, 5 g/d per cow of the appropriate MFS supplement was mixed into a 150 g/d ground corn topdress. During the covariate period all cows received the basal TMR with the ground corn topdress without MFS supplementation. All topdress was hand-mixed into the top 7 to 16 cm of TMR. Cows were housed in 2 ventilated tiestall barns with rubber mattresses bedded with sand. Cows were milked twice daily and barns and stalls were cleaned twice daily during milking.

**Table 1:** Ingredients and nutrient composition of diet

Item	% of DM
Ingredient	
Corn silage	36.40
Alfalfa silage	21.68
Ground corn	21.68
Canola meal	6.71
Corn gluten feed	6.02
Heat-treated soybean meal^1^	2.19
High RUP supplement^2^	0.91
Urea	0.32
Calcium carbonate	1.39
Rumen-protected Met^3^	0.059
Rumen-protected Lys^4^	0.032
Sodium sesquicarbonate	1.20
Potassium chloride	0.36
Potassium carbonate	0.27
Salt, white	0.28
Magnesium oxide	0.15
Biofix Select Pro^5^	0.052
Mineral oil	0.049
Complexed trace minerals^6^	0.049
Manganese sulfate	0.011
Zinc sulfate	0.0097
Vitamin E	0.0071
Monensin^7^	0.0071
Selenium yeast	0.0041
Biotin 2%	0.0036
Sodium selenite	0.0021
Vitamin A, D_3_	0.0009
Complexed copper chloride^8^	0.0009
Ethylenediamine dihydroiodide	0.0002
Nutrient composition	
Starch	30.12
aNDFom^9^	27.67
ADF	20.00
CP	16.91
Ash	8.23
Ether extract	4.19
Lignin	3.78
NE_L_ (Mcal/kg)	1.68

1 SoyPlus (Landus Cooperative).

2 ProVAAL AAdvantage (Perdue Agribusiness).

3 Smartamine M (Adisseo).

4 Ajipro-L G3 (Ajinomoto).

5 Biomin.

6 Availa-Dairy (Zinpro).

7 Rumensin 90 (Elanco Animal Health).

8 Intellibond (Micronutrients).

9 Neutral detergent fiber after amylase treatment and ashing.

Daily milk yields (a.m. plus p.m.) were recorded using electronic milk meters and recorded by DairyPlan software (GEA Farm Technologies). Milk samples were collected every Sunday a.m. and p.m. and sent for component analysis to Dairy Lab Services Inc. (Dubuque, IA). Daily DMI was recorded for each cow. Feed samples (TMR, forages, and concentrates) were collected on Saturdays. Dry matter was determined by oven drying for feed components and a Penn State shaker box was used to describe TMR particle size distribution. Feed samples were frozen and then composited by month and sent to Dairy One Forage Laboratory (Ithaca, NY) for nutrient analysis by wet chemistry techniques at the end of the study. Body weights were recorded on Monday and Tuesday after the p.m. milking at the start and end of the covariate period, then every 28 d until the end of the experiment. Body condition scores were recorded by the same 2 researchers during the same Tuesday weigh days using a 1 to 5 scoring system (Elanco Animal Health, 1996) and then averaged. If the difference between the assigned values of the 2 scorers was greater than 0.5 the scorers independently re-scored the cow. Averages of the values collected from the consecutive 2 d were used as the final BW and BCS for each time point. All daily values were averaged to weekly values by weeks in study and the covariate values were the average of 2 wk. Energy-corrected milk yield for each cow was calculated as 0.3246 × milk yield (kg/d) + 12.86 × fat yield (kg/d) + 7.04 × protein yield (kg/d) (NRC, 2001).

Data were analyzed using R (version 3.5; https://www.r-project.org/). The linear mixed effect model (LME) was

Y_ijklmno_ = T_i_ + W_j_ + L_k_ + D_l_ + K_m_ + P_n_ + C_o_ + e_ijklmno_,

where Y_ijklmno_ is the response variable, T_i_ = the fixed effect of treatment, W_j_ = the fixed effect of weeks on product, L_k_ = the fixed effect of parity, D_l_ = the DIM for each cow at the beginning of the treatment period, K_m_ = the fixed effect of enrollment sequence (i.e., group of cows added over time), P_n_ = the fixed effect of the average covariate production level per cow for the Y, T_j_ × W_i_ = the interaction between treatment and time, C_o_ is the random effect of individual cow, and e_ijklmno_ is the residual error term. No significant treatment by parity interactions were observed, hence the term was removed. Least squares means (LSM) were calculated via the emmeans package. Outliers were identified and removed by fitting a local polynomial regression (LOESS) for the response variable at the individual cow level and the residual for each value was calculated by subtracting the fitted value. Values with residuals that were greater or equal to the 0.975 quantile were considered outliers and removed. Each response variable was Box-Cox transformed before modeling to ensure a normal distribution and data points with residuals greater than 2.5 times the standard deviation were removed to improve the model fit. Data from the 3 cows that died later in the study were excluded from the analysis.

To evaluate the effect of starting DIM on the overall ECM gain, a generalized additive model with surrogate variable analysis was conducted in R:

Y_ijkl_ = *f*_1_(S_i_) + *f*_2_(D_j_) + *f*_3_(P_k_) + *f*_4_(C_l_) + ε_ijkl_,

where Y_ijkl_ = the ECM gain over 140 d, S_i_ = the surrogate variable representing the combined effect of treatment and starting DIM, D_j_ = the time (DIM) effect, P_k_ = the effect of covariate production level of ECM for each cow (penalized by the surrogate variable), C_l_ = the random effect of individual cows, and ε_ijkl_ = the residual error term. The final estimation was predicted using the constructed model with a covariate ECM = 40 kg/d (the average covariate ECM of this study) from a starting DIM of 43 to 145 d to represent our study. Means were compared by using 2 orthogonal contrasts: control versus MFS, and MFS1 versus MFS2. Values for *P* ≤ 0.05 were considered statistically significant, whereas values between 0.05 and ≤ 0.10 were considered as tendencies.

The baseline production means and statistics are shown in Table 2. No significant baseline differences among the treatment groups were observed except for SCC, where cows in the MFS2 group had significantly lower SCC than MFS1 group but no significant difference between control and MFS was observed (Table 2).

**Table 2:** Baseline data and the effect of microbial feed supplement on performance variables during experiment

Variable	Treatment^1^			Contrast *P*-value		Model *P*-value
	Control	MFS1	MFS2	Control vs. MFS	MFS1 vs. MFS2	Treatment × time^2^
Covariate period (baseline)						
Number of cows						
Primiparous	5	5	5	—	—	—
Multiparous	18	19	18	—	—	—
DIM	81 ± 26.3	85 ± 28.6	80 ± 24.8	0.94	0.32	—
Lactation no.	2.3 ± 1.1	2.4 ± 1.0	2.6 ± 1.3	0.30	0.42	—
Milk yield (kg/d)	39.2 ± 9.4	39.5 ± 7.5	38.2 ± 6.8	0.76	0.56	—
Milk composition						
ECM (kg/d)	41.7 ± 10.7	41.0 ± 7.1	39.4 ± 6.73	0.48	0.42	—
Fat (kg/d)	1.58 ± 0.58	1.5 ± 0.28	1.44 ± 0.32	0.24	0.49	—
Protein (kg/d)	1.22 ± 0.26	1.23 ± 0.21	1.2 ± 0.19	0.82	0.49	—
DMI (kg/d)	23.7 ± 3.4	23.4 ± 3.3	23.5 ± 3.3	0.70	0.99	—
Feed efficiency (ECM/DMI)	1.78 ± 0.50	1.76 ± 0.33	1.71 ± 0.31	0.58	0.54	—
Log_10_ (SCC)	4.76 ± 0.55	4.54 ± 0.52	4.94 ± 0.62	1.00	0.01	—
BW (kg)	670 ± 62.8	700 ± 79.9	674 ± 76.4	0.47	0.33	—
BCS	2.4 ± 0.39	2.4 ± 0.33	2.5 ± 0.42	0.42	0.59	—
Treatment period (LSM)						
Milk yield (kg/d)	34.7 ± 1.27	34.8 ± 1.22	36.7 ± 1.31	0.31	0.13	0.52
DMI (kg/d)	24.0 ± 1.0	23.8 ± 0.96	21.7 ± 1.04	0.14	0.04	0.69
Milk composition						
ECM (kg/d)	35.8 ± 1.32	37.2 ± 1.3	37.5 ± 1.34	0.16	0.86	0.007
Fat (%)	3.67 ± 0.18	3.79 ± 0.17	3.77 ± 0.18	0.48	0.90	0.79
Fat (kg/d)	1.27 ± 0.06	1.35 ± 0.06	1.31 ± 0.06	0.21	0.49	0.47
Protein (%)	3.30 ± 0.04	3.35 ± 0.04	3.32 ± 0.04	0.29	0.36	0.33
Protein (kg/d)	1.16 ± 0.04	1.17 ± 0.04	1.21 ± 0.04	0.31	0.30	0.71
Lactose (%)	4.69 ± 0.03	4.69 ± 0.03	4.69 ± 0.03	0.98	0.92	0.39
Lactose (kg/d)	1.66 ± 0.07	1.66 ± 0.07	1.74 ± 0.07	0.49	0.24	0.59
Other solids (%)	5.76 ± 0.03	5.79 ± 0.03	5.81 ± 0.03	0.10	0.68	0.046
TS (%)	12.79 ± 0.16	12.92 ± 0.15	12.87 ± 0.16	0.42	0.72	0.96
Log_10_ (SCC)	4.71 ± 0.10	4.80 ± 0.11	4.72 ± 0.11	0.59	0.55	0.15
MUN (mg/dL)	11.21 ± 0.40	11.48 ± 0.39	11.38 ± 0.4	0.42	0.77	0.46
Feed efficiency (ECM/DMI)	1.49 ± 0.08	1.53 ± 0.08	1.70 ± 0.1	0.08	0.06	0.41
BW (kg)	685 ± 9.7	682 ± 8.7	677 ± 9.9	0.48	0.64	0.049
BCS	2.8 ± 0.08	2.9 ± 0.08	2.7 ± 0.08	0.92	0.05	0.48

1 Control = no MFS supplement; MFS1 = 5 g/d Galaxis (Native Microbials Inc.); MFS2 = 5 g/d Galaxis Frontier (Native Microbials Inc.). Data ± SE.

2 Weeks in study.

No significant treatment effects were observed for milk yield, as the difference between MFS1 and MFS2 (Table 2) did not reach significance (*P* = 0.13). Likewise, the numerically lower DMI for MFS treatments compared with control did not reach significance (*P* = 0.14), but DMI was lower for MFS2 than for MFS1 (*P* = 0.04). Yield of ECM did not differ by treatments (*P* ≥ 0.16), but a significant treatment by time interaction was observed for ECM (Table 2). The general trend (Figure 1A) was for MFS1 and MFS2 to be greater than control over the first 13 wk of the study and for MFS2 to be greater than control or MFS1 during the remainder. The LSM over weeks on trial showed that ECM trended higher in MFS1 than control at wk 5 and trended higher in MFS2 than control at wk 19 (Figure 1A). Other solids content also showed a treatment by time interaction, where it trended higher in MFS1 and MFS2 than control at wk 2 and 6, respectively, and was significantly higher in MFS1 than control at wk 6 (Figure 1B). Mean BW showed a significant treatment by time interaction, but no trending or significant differences were observed in BW over each week (Figure 1C). The observations for ECM and other solids content are consistent with the overall results shown in Table 2, where numerical increases in performance variables such as milk yield and fat and protein contents were found in both MFS1 and MFS2, despite the lack of significance. Over the entire study period, a greater overall numerical difference in ECM was observed in MFS2 compared with control (+4.6%) and in MFS1 compared with control (+3.9%). When reviewing by weeks on study, a greater increase in ECM compared with control was observed in MFS1 before wk 14, when the lactation curves started to decline, whereas the MFS2 group showed greater ECM throughout the study, particularly after wk 14 (Figure 1A). The overall decrease in ECM after wk 14 suggests a transition to a different lactation stage where cows might have undergone a shift in metabolism and energy requirements. This shift at wk 14 in the MFS1 group also coincided with the changes in BW (Figure 1C).

**Figure 1:**
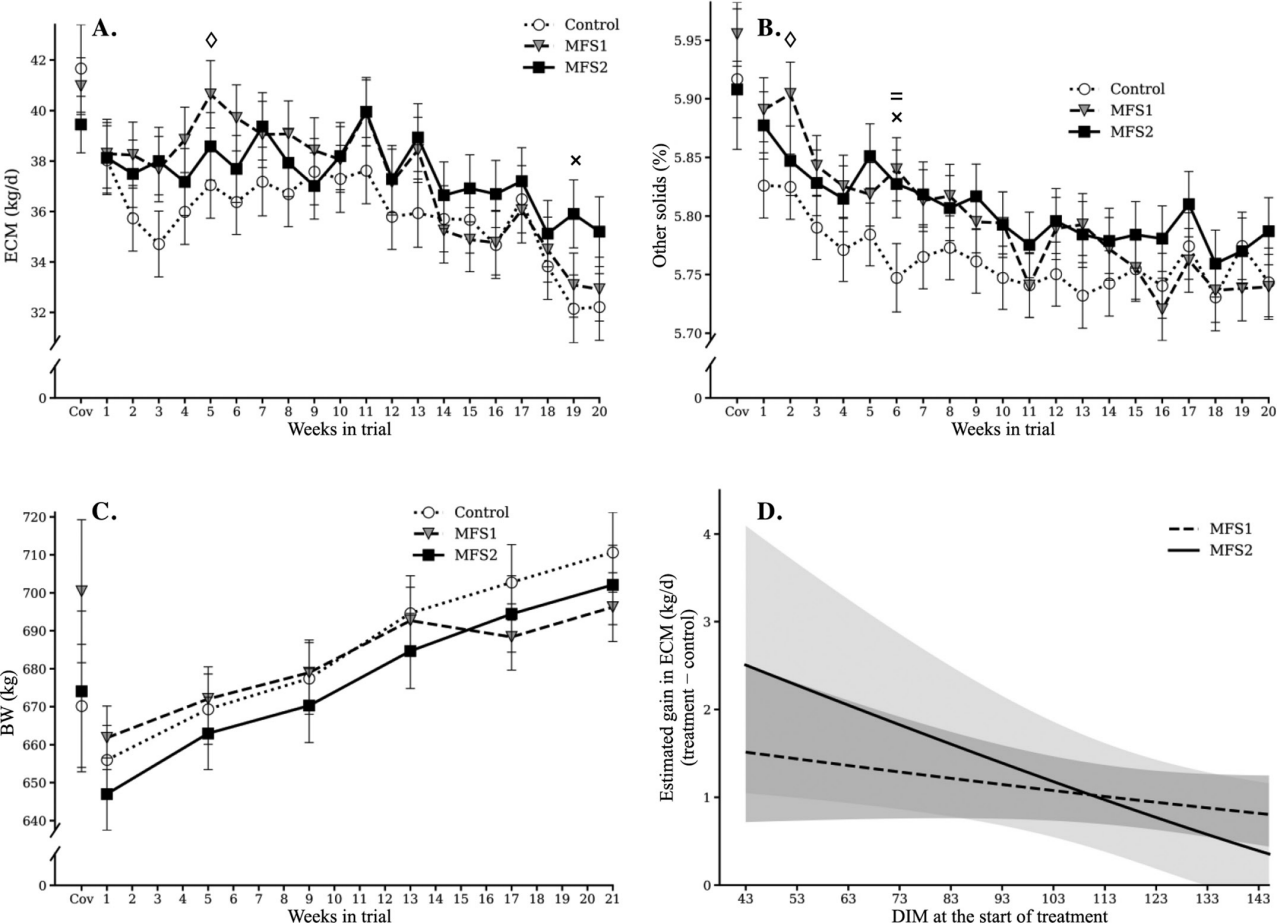
Production response variables with significant time by treatment interactions: ECM (A), milk other solids content (B), and BW (C), where ◊ denotes a *P*-value <0.1 between control and MFS1, × denotes a *P*-value <0.1 between control and MFS2, and = denotes a *P*-value <0.05 between control and MFS1. Panel D shows the starting DIM effect on ECM gain over the study period of 140 d. MFS1 = 5 g/d Galaxis (Native Microbials Inc.); MFS2 = 5 g/d Galaxis Frontier (Native Microbials Inc.). Error bars indicate SE.

Feed efficiency tended to be greater for MFS treatments than for control (*P* = 0.08), primarily because of the trend for MFS2 to be greater than MFS1 (Table 2). The microorganisms contained in MFS1 were evaluated previously by Goetz et al. (2021). In their study, the authors reported a significant treatment by time effect in FE, which was not detected in our study. However, both studies showed a similar overall numerical changes (not significant) in milk yield, ECM, fat and protein contents, and FE in the MFS1 group. The previous study also found that cows receiving MFS1 with milk yield less than 47 kg/d during the covariate period displayed an increase in ECM. Our findings align well with this result, where a 3.9% improvement in ECM was observed in the MFS1 group compared with control (Table 2). Goetz et al. (2021) also observed a numerical increase in DMI in the MFS1 group, whereas our study found that DMI was decreased in both MFS groups when compared with control. This difference might be because the cows used in the previous study were later in lactation (119 ± 38 DIM) than in our study. Based on our observations, different lactation stages could affect MFS performance (Figure 1D) due to changing metabolic energy needs throughout lactation (Ingvartsen and Moyes, 2013).

Although post hoc analysis revealed no significant BW differences among treatments at any week, the weight gain in MFS1 group clearly slowed between wk 13 and 17 (Figure 1C). This suggests that while the 2 microorganisms in MFS1 could improve rumen fermentation and support milk production, they may not be sufficient for the additional energy needed during a later lactation stage. This idea also is consistent with the observation of MFS2 treatment cows maintaining the same BW trajectory as the control group with no interruption between wk 13 and 17. Furthermore, we observed a negative correlation between starting DIM at enrollment and the ECM gain over the course of the study in both MFS groups (Figure 1D). Modeling revealed that, on average, a starting DIM of 43 d was predicted to improve ECM gain 2 and 7 times more than a starting DIM of 145 d in MFS1 and MFS2 group, respectively. However, the effect of the starting DIM was only significant in the MFS2 group (MFS2: *P* = 0.02, MFS1: *P* = 0.18). In addition, at the average starting DIM for MFS2 (82 d) in this study, the predicted ECM gain (+1.63 kg) in MFS2 group agreed with the ECM values reported in Table 2, supporting the accuracy of the model. The observation of the starting DIM effect on the improvements of production further suggests that the lactation stage and cow physiological shifts may have a significant impact on rumen fermentation and MFS efficacy. Future studies should attempt to group cows more closely based on starting DIM and with sufficient cows in each stage to detect differences in response.

No significant differences were detected in the number of cows diagnosed with mastitis among the treatments (Fisher's exact test *P*-value = 0.88). Including the 2 cows that died from toxic mastitis, there were 6 control cows, 6 MFS1 cows, and 5 MFS2 cows that had mastitis, representing a total of 9, 8, and 9 incidences from each respective group. The average days per incident was 13 d for control, 11 d for MFS1, and 12 d for MFS2. Thus, the mastitis events were similar among groups with no treatment association.

Overall, FE tended to be improved, especially by MFS2. In addition, the use of rumen native MFS increased ECM yield at wk 5 and 19 of supplementation in lactating Holstein cows without a negative impact on BW. One weakness of our study is the relatively large range in starting DIM of the cows enrolled. With the observation that production improvement was negatively correlated with the starting DIM, the cows enrolled at a later DIM would have a diminished response, thus requiring an increase in the number of cows to meet the same statistical power. In addition, the study had a long enrollment period, which resulted in some cows finishing the study in winter months, whereas some finished in summer months. This increased variability may have contributed to the reduced statistical power. Although the supplementation of both MFS to lactating cows was beneficial, more research is needed to determine differential effects of MFS at different lactation stages.
